# Anti-Fading Media for Live Cell GFP Imaging

**DOI:** 10.1371/journal.pone.0053004

**Published:** 2012-12-21

**Authors:** Alexey M. Bogdanov, Elena I. Kudryavtseva, Konstantin A. Lukyanov

**Affiliations:** Shemyakin-Ovchinnikov Institute of Bioorganic Chemistry, Moscow, Russia; The Beatson Institute for Cancer Research, United Kingdom

## Abstract

Photostability is one of the most important characteristic of a dye for fluorescence microscopy. Recently we demonstrated that vitamins present in imaging media dramatically accelerate photobleaching of Enhanced Green Fluorescent Protein (EGFP) and many other green fluorescent and photoactivatable proteins. Here we tested all vitamins of commonly used media (such as Dulbecco's Modified Eagle Medium, DMEM) one-by-one and found that only two vitamins, riboflavin and pyridoxal, decrease photostability of EGFP. Thus, DMEM without riboflavin and pyridoxal can be used as an imaging medium, which ensures high photostability of GFPs at the expense of minimal biochemical disturbance. Then, we tested some antioxidants and found that a plant flavonoid rutin greatly enhances photostability of EGFP during live cell microscopy. In complete DMEM, rutin increased EGFP photostability up to the level of vitamin-depleted DMEM. Moreover, being added to vitamin-depleted DMEM, rutin was able to further suppress EGFP photobleaching. Potentially, new medium formulations can be widely used for fluorescence microscopy of GFP-expressing cells and model multicellular organisms in a variety of imaging applications, where photostability represents a challenge.

## Introduction

Green fluorescent protein (GFP) from the jellyfish *Aequorea victoria*, its homologs from diverse marine animals, and their numerous mutants, has become extremely popular for fluorescence labeling of living cells [1]. Brightness and photostability are key characteristics for successful applications of fluorescent probes in microscopy. High photostability is especially important for prolonged time lapse imaging extensively used to track cell movements, mobility of intracellular organelles and structures, changes in distribution of target proteins upon stimuli or in cell cycle, etc. Reconstruction of 3D images also requires extensive illumination of objects at different z-planes and often suffers from insufficient photostability of a probe. In addition, single-molecule-sensitive techniques, such as fluorescence correlation spectroscopy [2] and recently introduced super-resolution imaging [3] would benefit greatly from lowered photobleaching. In particular, Photoactivation Localization Microscopy (PALM) and related techniques are based on molecule-by-molecule photoconversion and following detection of single molecules of photoactivatable fluorescent proteins [4]. For PALM, photostability of the activated state is vital for high precision of localization of single molecules since it is proportional to number of photons emitted from individual fluorophores. To increase the photostability of fluorophores, a number of anti-fading media that decrease photobleaching of various chemical dyes have been developed [5]. However, they are applicable only to fixed samples. Another highly efficient way to reduce photobleaching is to remove molecular oxygen from the medium, e.g., using Oxyrase reagent [6]. This method can be applied to live cells, but cell physiology would be strongly disturbed by the anaerobic conditions produced.

Recently we discovered ability of various green FPs to act as light-induced electron donors in reactions with appropriate electron acceptors [7]. As a result of this photochemical reaction, GFPs convert from green to red fluorescent state (“oxidative reddening”). Importantly, biological redox-active molecules such as flavins, flavoproteins, and cytochromes can support oxidative reddening of GFPs. Moreover, it occurs in live cells expressing GFP without addition of exogenous oxidants. It was then realized that oxidative reddening accounts for a considerable (sometimes major) part of GFP photobleaching observed during living cells microscopy [8]. We demonstrated that exclusion of potentially redox-active components from the cell medium allows to reduce photobleaching dramatically [8]. In particular, commonly used cell medium DMEM (Dulbecco's Modified Eagle Medium) without riboflavin provides several-fold improvement of EGFP photostability. DMEM without all vitamins (commercial name DMEM^gfp^, referred to as DMEM-V in [8]) even further enhances GFP photostability. Vitamin-depleted media for imaging represent as efficient way to reduce GFP photobleaching. However, this method has obvious drawbacks. First, the lack of vitamins can have deleterious effects on cell physiology. Second, the use of poor media is inapplicable for *in vivo* imaging in whole model organisms.

The present work was focused on development of media for high GFP photostability in live cell imaging with two main goals: (i) to establish medium composition which provides maximal GFP photostability at the expense of minimal biochemical disturbance, and (ii) to search for compounds, which can be added to improve GFP photostability. As a result, we succeeded in development of media formulations with minimal vitamin depletion and maximal photostability of EGFP.

## Results and Discussion

We previously showed that removing all vitamins from DMEM results in dramatic enhancement of photostability of GFPs during live cell imaging [8]. To find vitamins that do affect GFP photostability we tested changes of EGFP photostability after addition of each vitamin to DMEM^gfp^ (DMEM without vitamins). Several DMEM^gfp^-based media containing separately each of 7 compounds (riboflavin, thiamine-hydrochloride, pyridoxal-hydrochloride, nicotinamide, folic acid, choline-chloride, D-Ca-pantothenate) were prepared. We used the same concentrations as formulated in DMEM. L-inositol was excluded from testing as a molecule which does not have evident redox properties.

**Figure 1:**
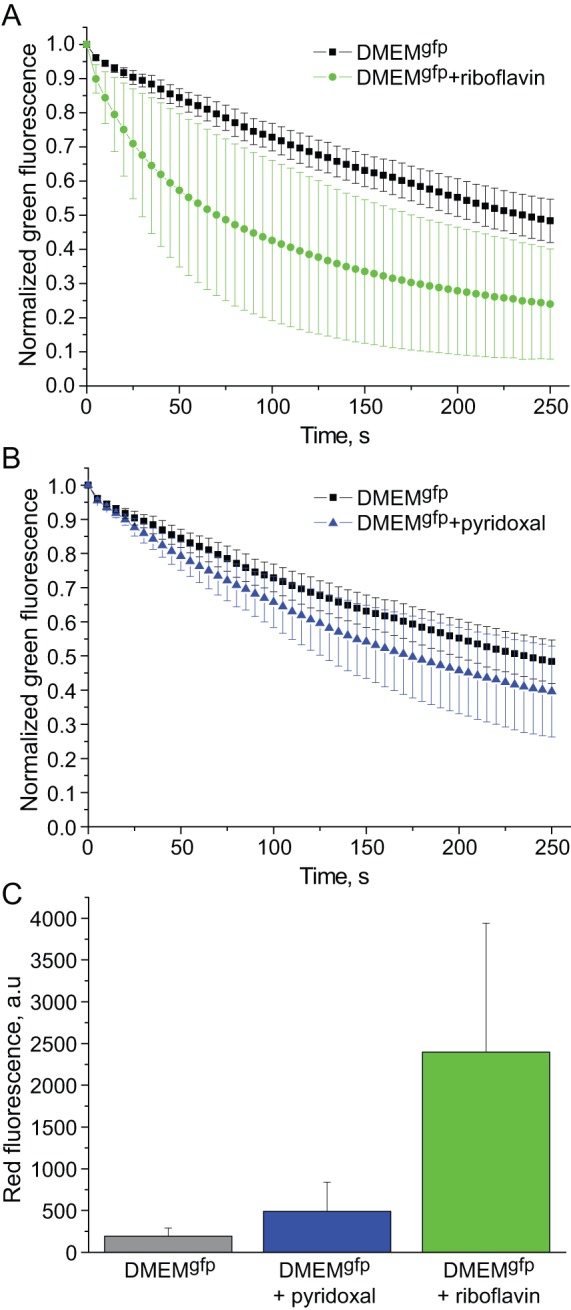
Riboflavin and pyridoxal decrease EGFP photostability. Graphs show bleaching of EGFP fluorescence in live HEK293T cells in DMEM^gfp^ (DMEM lacking vitamins) (black squares) or in DMEM^gfp^ supplemented with riboflavin (**A**, green circles) or pyridoxal (**B**, blue triangles). Fluorescence intensities in individual cells are background subtracted and normalized to maximum (initial) value in each cell. Standard deviation values (n = 15–20 cells in a representative experiment out of three independent experiments) are shown.

**Figure 2:**
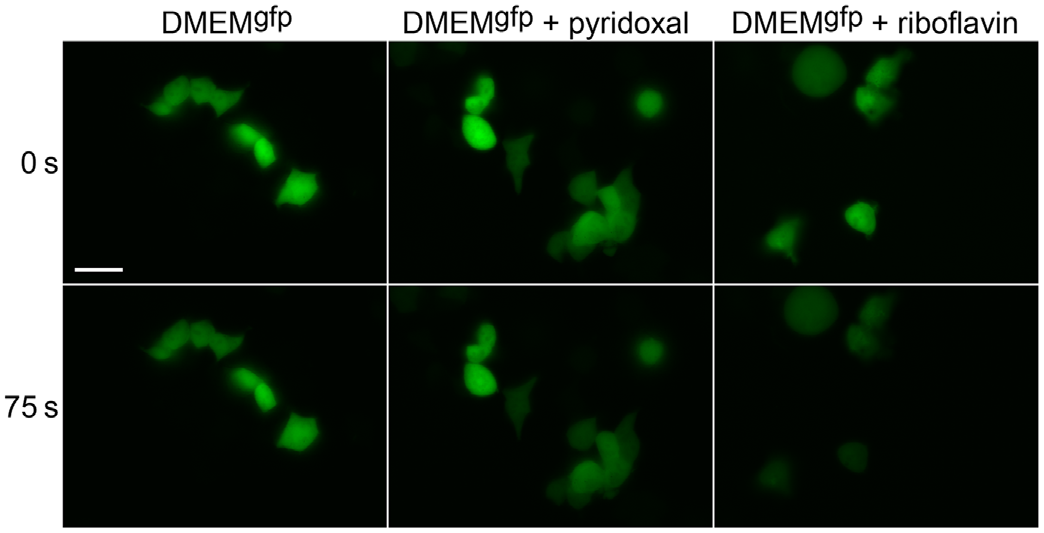
Fluorescence microscopy of live HEK293T cells transiently expressing EGFP. Representative raw images of cells in green channel used to compose graphs in Figure 1A and B are shown. Cells were imaged in DMEM^gfp^ (left), DMEM^gfp^ with pyridoxal (middle), and DMEM^gfp^ with riboflavin (right) before (upper row) and after (bottom row) 75 s bleaching with intense blue light. Scale bar 20 µm.

HEK293T cells transiently transfected with EGFP were grown in complete DMEM. Before imaging, DMEM was changed to DMEM^gfp^ or DMEM^gfp^ supplemented with one of the above mentioned vitamins. Among tested compounds only riboflavin and pyridoxal exerted negative effect on EGFP photostability (Figs. 1 and 2), whereas another 5 vitamins did not change EGFP bleaching curves compared to DMEM^gfp^ (not shown). Notably, appearance of red signal during EGFP photobleaching was several-fold higher in the presence of pyridoxal or riboflavin compared to DMEM^gfp^ (Fig. 1C). Thus, we concluded that pyridoxal- or riboflavin-enhanced photobleaching is related to EGFP oxidative reddening phenomenon [7], [8].

Influence of pyrodoxal was significantly lower than that of riboflavin, in spite of 20-fold higher concentration of pyrodoxal (20 µM pyridoxal *versus* 1 µM riboflavin). Under current experimental conditions addition of riboflavin (0.4 mg/L) to cell medium led to about 3.3-fold bleaching half-time decrease, while pyridoxal (4 mg/L) gave only 1.5-fold effect. Thus, concentration-normalized effect of riboflavin on EGFP photostability is approximately 40 times higher than that of pyridoxal.

**Figure 3:**
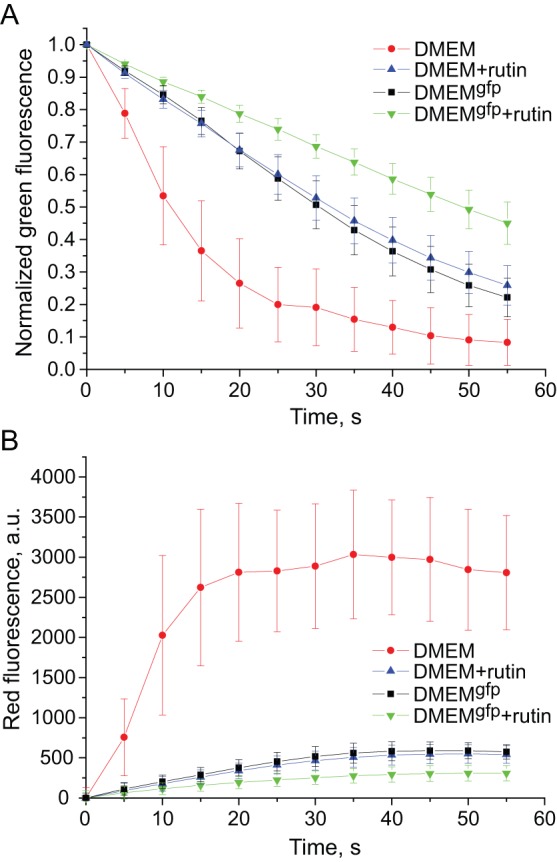
Influence of rutin on EGFP photobehavior. Graphs show bleaching of green fluorescence (**A**) and simultaneous appearance of red fluorescence (**B**) in EGFP-expressing live HEK293 cells maintained in DMEM (red circles), DMEM with rutin (blue triangles), DMEM^gfp^ (black squares), or DMEM^gfp^ with rutin (inverted green triangles). Green fluorescence intensities in individual cells (A) are background subtracted and normalized to maximum (initial) value in each cell. Red fluorescence intensities in the same cells (B) are background subtracted and corrected for initial green fluorescence in respective cells. _Standard deviation values (n = 15–20 cells in a representative experiment out of five independent experiments) are shown.

**Figure 4:**
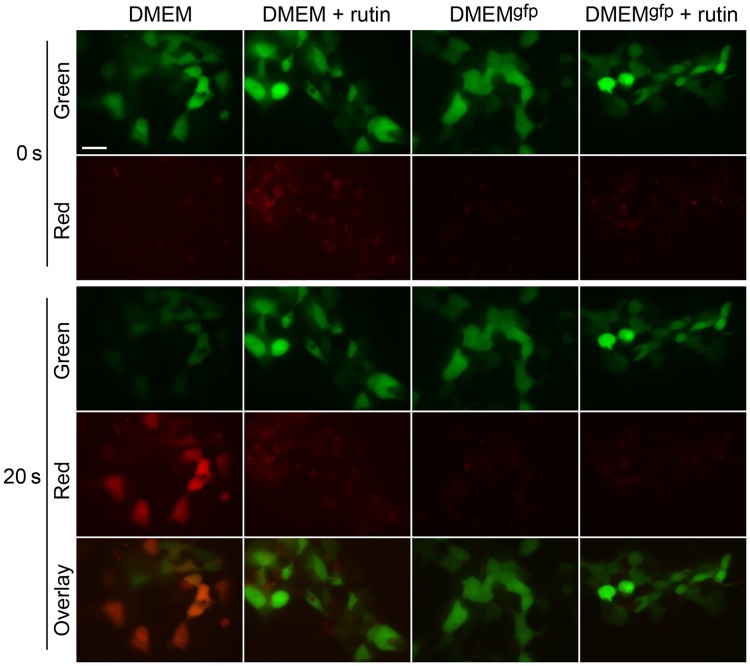
Bleaching and reddening of EGFP-expressing cells in media with and without rutin. Representative raw images in green and red channels used to compose graphs in Figure 3 are shown. Cells were imaged in media designated above the images before (upper pannels) and after (bottom panels) 20 s bleaching with intense blue light. Scale bar 20 µm.

Then, we hypothesized that enhanced GFP photostability can be achieved by addition of some reducing agents or antioxidants preventing GFP oxidative photoconversion. We tested three compounds of different structure: glutathione, ascorbate and rutin (plant flavonoid glycoside also known as vitamin P). Among them only rutin demonstrated positive effect on EGFP photostability. Being added to the standard DMEM 30 min before imaging, rutin ensured dramatic increase of EGFP photostability up to the level observed in vitamin-depleted DMEM^gfp^ (Fig. 3A and Fig. 4). Moreover, addition of rutin to DMEM^gfp^ resulted in even further enhanced EGFP photostability (about 1.5-fold compared to DMEM^gfp^). It was found that rutin greatly suppresses green-to-red photoconversion of EGFP in both DMEM and DMEM^gfp^ media (Fig. 3B and Fig. 4). Thus, we concluded that mechanism of action of rutin on GFP photostability is inhibition of oxidative reddening.

Interestingly, effect of rutin on EGFP photobleaching was observed in live cells imaging only, but not in *in vitro* experiments. For example, rutin did not change rates of photobleaching and oxidative reddening of EGFP immobilized on a metal-affinity resin beads and placed in DMEM or DMEM^gfp^ media (not shown). This indicates that influence of rutin on photochemical GFP behavior is mediated by intracellular biochemical pathways. Rutin and its analogs were previously described as efficient scavengers of radicals [9], [10]. In addition, it was recently demonstrated that rutin reacts with light-excited flavins [11]. We believe that these antioxidant properties of rutin underlie its ability to suppress oxidative reddening of GFP, although the exact mechanisms of this process remains unclear and calls for further studies.

To conclude, our studies on influence of various redox-active compounds on GFP photobleaching resulted in two main findings. First, it was shown that only two components, riboflavin and pyridoxal, in widely used DMEM medium considerably decrease EGFP photostability. Thus, we suggest a minimally depleted medium (DMEM with no riboflavin and pyridoxal) as an imaging medium for low-photobleaching microscopy of GFP-expressing cells. Whereas DMEM^gfp^ was found to have no obvious deleterious effects on cell morphology, motility and proliferation for several days [8], the new formulation depleted in only two vitamins can be used even more safely. Second, we found that the plant flavonoid rutin is able to enhance intracellular GFP photostability dramatically. Rutin increased EGFP photostability in rich media, and even additionally improved results of vitamin-depleted low-photobleaching medium DMEM^gfp^. Importantly, rutin-mediated enhancement of GFP photostability is potentially applicable not only for cell cultures, but also for multicellular model organisms, where vitamin-depleted media can not be used.

## Materials and Methods

### Cell culture

Human embryonic kidney HEK293T cell line was grown at 37°C in 5% CO_2_ atmosphere in DMEM (PanEco) supplemented with 10% FBS (Sigma). Cells were transiently transfected with pEGFP-C1 vector (Clontech) using FuGene 6 reagent (Roche) in accordance to the manufacturer's recommendations. Cells were analyzed by fluorescence microscopy at room temperature 24–48 h post transfection.

### Fluorescence microscopy

Leica AF6000 LX imaging system with a Photometrics CoolSNAP HQ CCD camera was used for fluorescence microscopy of live cells. A 120W HXP short arc lamp (Osram) was used as a light source. Green and red fluorescence images were acquired using 60x oil immersion objective and standard filter sets: GFP (excitation BP470/40, emission BP525/50) and TX2 (excitation BP560/40, emission BP645/75). Photobleaching and oxidative photoconversion was monitored in time-lapse imaging in the green and red channels at low light intensity combined with 5-s exposures to blue light of maximum intensity (GFP filter set). The intensity of bleaching light varied from 0.5 to 1.9 W/cm^2^ because of long-term “aging” of lamp and optical fiber, but it was constant within each comparative experiment. The total power of the excitation light after objective was measured using a Laser Power Meter LP1 (Sanwa). Images were acquired and quantified using Leica LAS AF software. Graphs were generated using Origin software.

### Imaging media

Before fluorescence microscopy, the growth medium DMEM was replaced under sterile conditions with modified media for imaging and incubated for 30 min at 37°C in a CO_2_-incubator. DMEM^gfp^ (Evrogen) lacking all vitamins was used as a base to test influence of each vitamin on EGFP photostability. Individual vitamins were added to DMEM^gfp^ at final concentrations corresponding to complete DMEM (riboflavin 0.4 mg/L (Sigma), thiamine-hydrochloride 4 mg/L (AppliChem), pyridoxal-hydrochloride 4 mg/L (AppliChem), nicotinamide 4 mg/L (DiaM), folic acid 4 mg/L (AppliChem), choline-chloride 4 mg/L (DiaM), D-Ca-pantothenate 4 mg/L (AppliChem)).

Antioxidants glutathione (Sigma), ascorbate (Ozon Pharm) and rutin (Acros) were added to DMEM or DMEM^gfp^ 30 min before imaging at final concentrations 1 mM, 100–1000 µM, and 16 µM (10 mg/L), respectively. It was found that rutin undergo degradation in water solutions in a 20–30 h timescale. So, freshly prepared rutin solutions were used in all experiments.
